# A Soft Pneumatic Two-Degree-of-Freedom Actuator for Endoscopy

**DOI:** 10.3389/frobt.2021.768236

**Published:** 2021-11-11

**Authors:** Gilles Decroly, Pierre Lambert, Alain Delchambre

**Affiliations:** ^1^ TIPs Dpt CP 165/67, Université Libre de Bruxelles, Brussels, Belgium; ^2^ BEAMS Dpt CP 165/56, Université Libre de Bruxelles, Brussels, Belgium

**Keywords:** soft robotics, pneumatic actuator, endoscopy, minimally invasive surgery, steerable catheter

## Abstract

The rise of soft robotics opens new opportunities in endoscopy and minimally invasive surgery. Pneumatic catheters offer a promising alternative to conventional steerable catheters for safe navigation through the natural pathways without tissue injury. In this work, we present an optimized 6 mm diameter two-degree-of-freedom pneumatic actuator, able to bend in every direction and incorporating a 1 mm working channel. A versatile vacuum centrifugal overmolding method capable of producing small geometries with a variety of silicones is described, and meter-long actuators are extruded industrially. An improved method for fiber reinforcement is also presented. The actuator achieves bending more than 180° and curvatures of up to 0.1 mm^−1^. The exerted force remains below 100 mN, and with no rigid parts in the design, it limits the risks of damage on surrounding tissues. The response time of the actuator is below 300 ms and therefore not limited for medical applications. The working space and multi-channel actuation are also experimentally characterized. The focus is on the study of the influence of material stiffness on mechanical performances. As a rule, the softer the material, the better the energy conversion, and the stiffer the material, the larger the force developed at a given curvature. Based on the actuator, a 90 cm long steerable catheter demonstrator carrying an optical fiber is developed, and its potential for endoscopy is demonstrated in a bronchial tree phantom. In conclusion, this work contributes to the development of a toolbox of soft robotic solutions for MIS and endoscopic applications, by validating and characterizing a promising design, describing versatile and scalable fabrication methods, allowing for a better understanding of the influence of material stiffness on the actuator capabilities, and demonstrating the usability of the solution in a potential use-case.

## 1 Introduction

In the medical field of minimally invasive surgery (MIS) and endoscopy, the rise of soft robotics, using materials of similar softness to biological tissues, opens many new opportunities (Cianchetti et al., 2018; Gorissen et al., 2017). Soft actuated catheters could become an alternative to current steerable catheters, by minimizing the risk of damage to surrounding tissues while enhancing the possibilities to navigate in a confined space and to reach remote locations (Blanc et al., 2017). In particular, fluidic or pneumatic actuators present the advantage to be safe, since they do not require rigid parts nor electrical voltage, to be lightweight, to allow the reduction of the number of parts needed for a given movement, and to be MRI compatible (De Greef et al., 2009).

Since the first design presented by Suzumori (Suzumori et al., 1997), numerous pneumatic actuators for MIS and endoscopy have been presented, but none has reached clinical application yet. Runciman et al. reviewed soft robotics devices for MIS, including mainly pneumatic actuators. The device description is generally limited to the actuated segment, which is intended to be used as a steerable segment for endoscopes or catheters. Among the variety of designs described in the literature, diameters as small as 1 mm (Gorissen et al., 2018) have been reported. However, actuators having external diameters below 10 mm are generally only able to bend in one direction (one pressurized channel) (Ikuta et al., 2012; Gorissen et al., 2018) or do not include any working channel (Gorissen et al., 2018). At a larger scale (external diameters larger than 10 mm), Garbin et al. integrated pneumatic actuators in a complete low-cost endoscope system (Garbin et al., 2019). Similarly, the STIFF-FLOP project developed functional multi-module soft manipulators for MIS (Abidi et al., 2018; Brancadoro et al., 2019). Toward miniaturized actuators for steerable catheters, Sun et al., Zhang et al., and Chauhan et al. proposed three similar and promising designs. They embedded three pressurized channels and a working channel, which can facilitate bending in every direction (Sun et al., 2016; Zhang et al., 2019; Chauhan et al., 2021). Recently, Gopesh et al. (2021) presented a steerable catheter having an external diameter of 0.9 mm and made of a soft fluidic steerable tip actuated by four circumferential pressurized channels, assembled with a 1.6 m long Pebax catheter. They demonstrated the possibility to use it for endovascular surgical procedures on *ex vivo* silicone models of the human vasculature, and in *in vivo* experiments in porcine (Gopesh et al., 2021). However, except for this groundbreaking example, the integration of such actuators in steerable catheters remains poorly demonstrated. Moreover, the influence of the main material properties on their mechanical capabilities has never been studied experimentally. The possibility to mechanically program an actuator by modulating the material stiffness has been demonstrated (Ke et al., 2021), but it relies on a specific design and mathematical model. Finally, manufacturing remains an important challenge, and there is a need for a highly repeatable, fast, low-cost, and versatile model able to produce small geometries (Runciman et al., 2019).

In this work, we present an optimized 6 mm diameter pneumatic actuator embedding a working channel and having two degrees of freedom in bending, i.e., able to bend in every direction. To address the manufacturing challenges, two innovative fabrication methods have been investigated: a versatile vacuum centrifugal overmolding method able to produce small geometries with a variety of silicone materials has been developed and industrially extruded actuators have been studied. An improved method for fiber reinforcement is also presented. To provide a better understanding of the actuator behavior, the mechanical capabilities (curvature, blocking force, working space, multi-channel actuation, and response time) are characterized experimentally and analyzed using a simple Euler–Bernoulli model. An emphasis is placed on the study of the influence of material stiffness on mechanical capabilities. Finally, a proof-of-concept of a 90 cm long steerable catheter is developed, and its potential for endoscopy is demonstrated in a bronchial tree phantom.

## 2 Design

**FIGURE 1:**
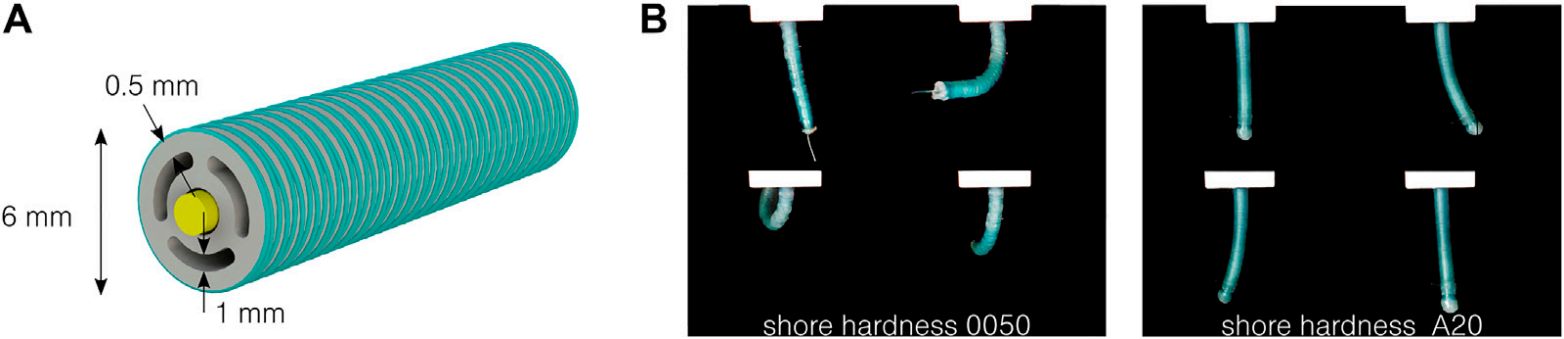
Design of the two-degree-of-freedom fluidic actuator. **(A)** Cross-section of the actuator, composed of a silicone body in gray, a thread or endoscopic tool acting as a neutral fiber in yellow, and fiber reinforcement in green. **(B)** The actuator bends in every direction by pressurizing the different circumferential channels, here at 0.5 bar with two different silicones.

In our previous work, a one-degree-of-freedom pneumatic actuator (i.e., having one pressurized channel and able to bend in one direction) has been designed, characterized, and modeled (Decroly et al., 2020). This allowed us to identify and validate the key design parameters of the actuator, namely, the ratio between the pressure channel area and the overall actuator cross-sectional area. Based on these results, and inspired by similar designs (Sun et al., 2016; Zhang et al., 2019; Chauhan et al., 2021), an optimized actuator is presented in Figure 1A. The soft silicone body includes four lumens: three circumferential lumens, which can be pressurized to generate bending in every direction, and a central lumen, allowing to carry medical tools. The leads of a camera or the tool in the central channel also act as neutral fibers of the actuator. The external fiber reinforcement is composed of a double helix, using a 0.2 mm diameter polyester thread with a pitch of 0.5 mm. Its main role is to limit the radial expansion, without increasing the bending stiffness of the actuator (Wang et al., 2017). The outer diameter of the actuator without the reinforcement is 5 mm, and 6 mm after the reinforcement. The external wall and pressurized channel thicknesses are, respectively, 1 and 0.5 mm. The ends of the pressurized channels are sealed. A thread is used in the central channel as a neutral fiber. As demonstrated in Figure 1B, the actuator bends in every direction, by pressurizing the opposite channel, or a combination of channels. Note that no elongation of the actuator can be obtained with the presented design, since the leads placed in the central channel limit act as strain-limiting fibers. The actuator length ranges from 20 to 50 mm. It is intended to be used as an active segment for steerable catheters, which can reach a length up to 2 m (Runciman et al., 2019).

## 3 Manufacturing

Two innovative fabrication methods are presented in this work. The challenges of the design lie in the thin geometries (as thin as 0.5 mm), the need to have completely bubble-free actuators to avoid leakage or failure, and the high aspect ratio (5–10 for the actuator and up to more than 400 for a steerable catheter). An innovative vacuum centrifugal overmolding process is presented and allows for the fabrication of actuators made of different silicones. Alternatively, as already proposed by Suzumori (Suzumori, 1989; Suzumori et al., 1997), the constant cross-sectional geometry makes the design suitable for extrusion. The properties of the materials used in this study are presented in Table 1.

**TABLE 1:** Silicones used in this study. The values are extracted from the datasheets^6^. Young’s modulus is estimated as the stress at 100% elongation. Ecoflex and Dragon Skin are used for molding and PR410/40 for extrusion.

	Specific gravity (g/cm^3^)	Shore hardness	Tensile strength (MPa)	Stress at 100% (MPa)	Elongation at break (%)	Uncured viscosity (Pa s)
Ecoflex 0030 (E30)	1.07	00–30	2.41	0.069	900	3
Ecoflex 0050 (E50)	1.07	00–50	2.17	0.083	980	8
Dragon Skin 10 (D10)	1.07	10A	3.28	0.151	1,000	23
Dragon Skin 20 (D20)	1.08	20A	3.79	0.338	620	20
Extruded PR410/40 (Ext)	1.11	40A	8.2		620	n.a.

### 3.1 Vacuum Centrifugal Overmolding

**FIGURE 2:**
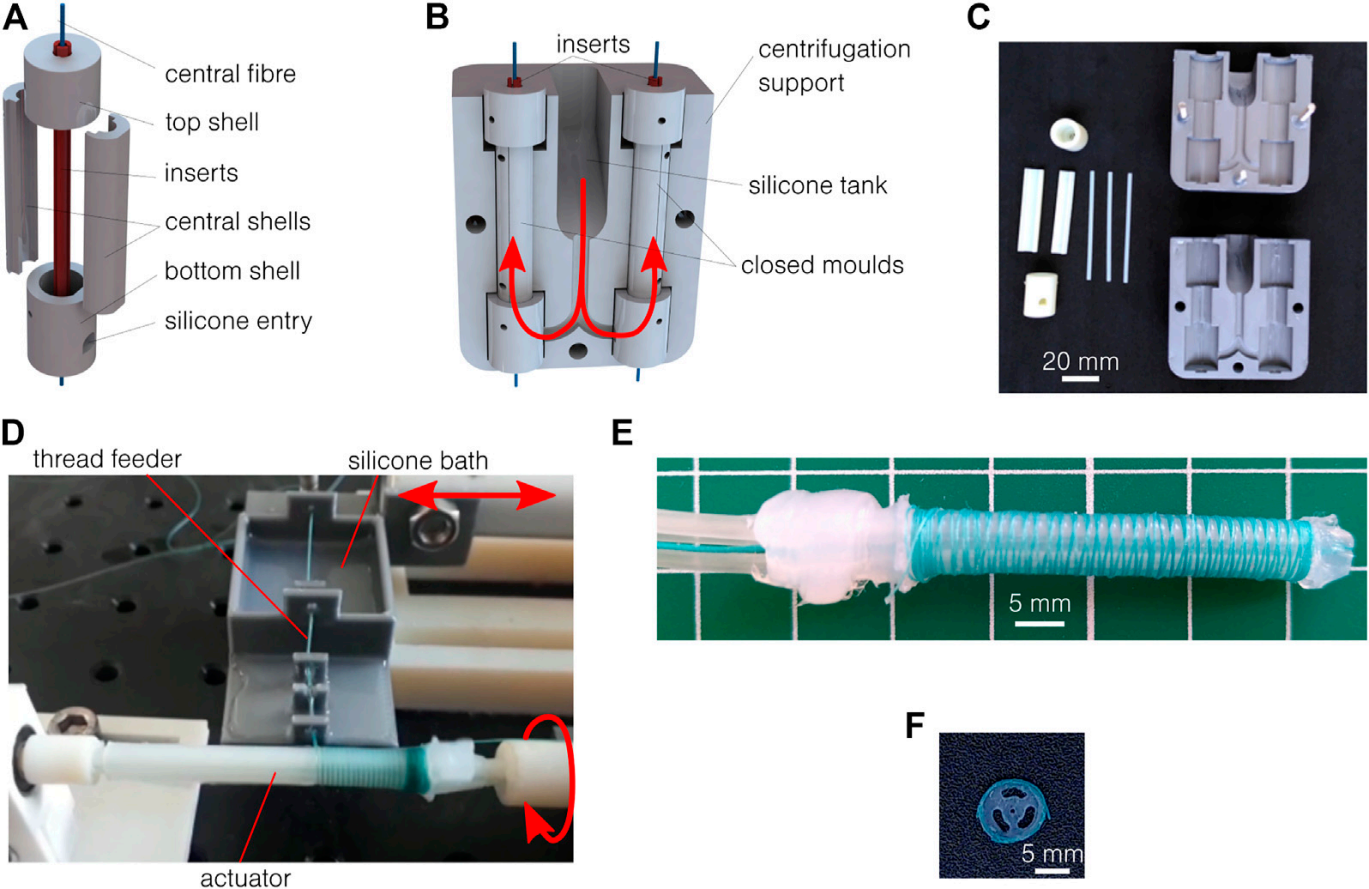
Vacuum centrifugal overmolding process. **(A)** The mold is made of seven parts plus the central fiber to be overmolded. **(B)** The closed mold is placed in a two-part support, which includes a tank for the uncured silicone. When centrifuged, the silicone flows along the red arrows. **(C)** 3D printed molds and supports. **(D)** Setup for fiber reinforcement. **(E)** Results of the fabrication process after reinforcement, sealing, and assembly. **(F)** The cross-section respects the designed geometry.

This new process is inspired by Sun et al. (2016) and allows for the fabrication of geometries as thin as 0.5 mm on a length of 50 mm and with silicone of high viscosity (up to 23 Pa s in this work). The molds are made of seven parts, as illustrated in Figure 2A: four for the external shell and three inserts for the lumens. The parts are 3D printed using polyjet technology. A 0.5 mm diameter polyester thread is directly placed in the mold (under tension) as a central fiber to be overmolded. Once closed (Figure 2B), those molds are placed in a two-part support for centrifugation, containing a tank for the silicones and ensuring a good silicone filling. All the parts are shown in Figure 2C. The two-part silicone is first mixed manually and placed under vacuum to remove trapped air bubbles and then poured into the silicone tank. The mold and support are placed in a centrifuge, under vacuum. The acceleration is approximately 900 m/s^2^ for 1 min. Thanks to the centrifugal force, the silicone flows through the channels and enters the molds from the bottom, as shown in red in Figure 2B. The air is expelled along the inserts. The reticulation lasts several hours, depending on the material. The samples are then released from the mold. A little bit of post-processing is required to clean the sample from cured thin leakages (flashing). The external shell and central insert can be removed, but the centrifugal inserts must remain for the fiber reinforcement process. To add the reinforcement, the actuator is placed on the axis of a stepper motor, while a thread supplier is placed on a linear motor, parallel to the actuator. The setup is shown in Figure 2D. As an improvement compared to previously reported and similar setups (Sun et al., 2016), the thread is here continuously soaked in a silicone bath before the reinforcement. This additional silicone cures directly around the thread, ensuring a perfect adhesion with the main body, while avoiding the manual addition of an external silicone layer afterward. This increases the final diameter to (5.97 ± 0.17) mm, approximated to 6 mm in this work. A 0.2 mm diameter polyester thread is used, with a controlled pitch of 0.5 mm. Several steps remain to seal and connect the actuator: remove the inserts, fix the central thread on both ends to allow it to act as a neutral fiber, seal the distal end of the radial pressurized channels, and connect and seal the tubing to the pressure source on the proximal end. Silicone glue is used here. An example of an obtained actuator is shown in Figure 2E, and the cross-section is shown in Figure 2F. The process showed similar results to the four Ecoflex and Dragon Skin silicones presented in Table 1.

This innovative process, thanks to vacuum centrifugation, allows for the fabrication of extremely thin geometries with different silicones having a large range of uncured viscosity and stiffness. Furthermore, the new reinforcement process, using threads soaked in silicone, allows controlling the reinforcement pitch and a perfect adhesion of the thread. However, the inserts are subject to bending if not placed perfectly, which can induce imperfections and inter-channel leakage for some samples. Their release can also induce defects. On the 22 actuators produced with the final process, six were to be discarded. The complete process also requires many manual steps and takes approximately one day for a batch of 16 samples.

### 3.2 Extrusion

**FIGURE 3:**
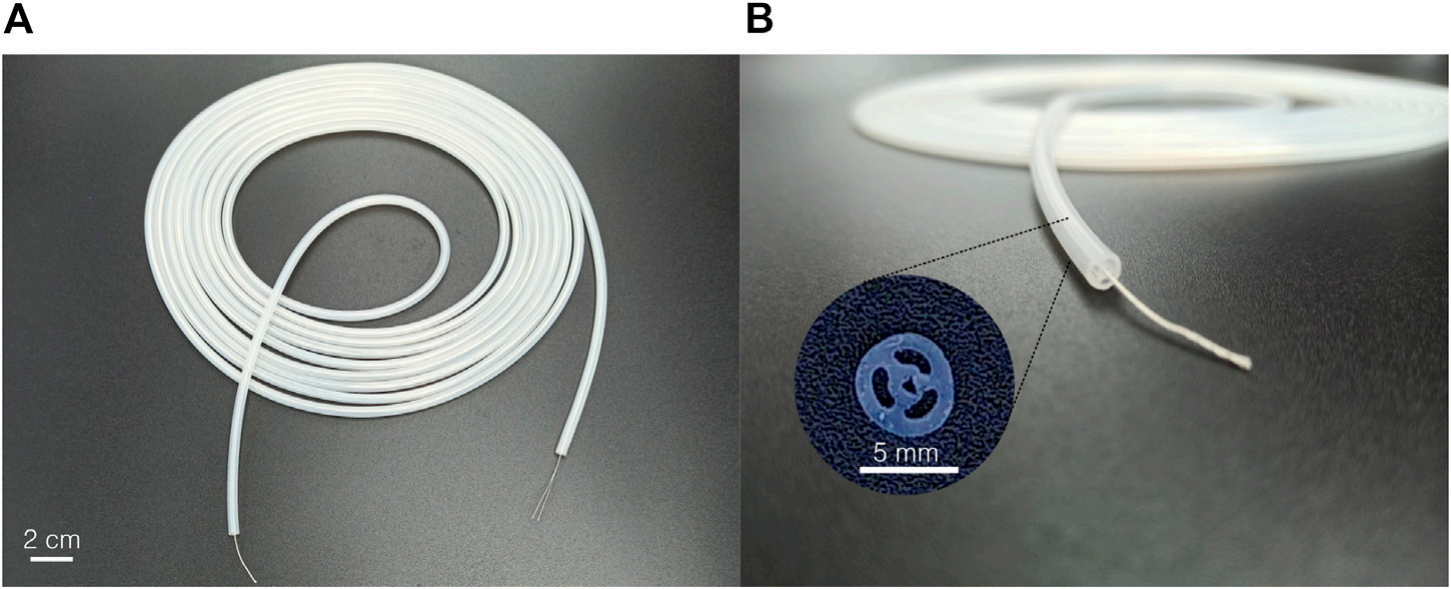
Industrially extruded hardness 40A silicone sample. **(A)** The 5 m long silicone tube is coextruded around a central thread. **(B)** The cross-section respects the dimension but shows small imperfections.

As an alternative solution and toward future industrialization, extrusion has been considered an alternative fabrication process for the design presented in Figure 1. Even if well known and often considered (Runciman et al., 2019), few works reported the use of extrusion to produce similar constant cross-section and multi-lumen actuators (Suzumori, 1989; Suzumori et al., 1997). Extrusion presents several advantages: it is an industrial and scalable process, commonly used for the production of medical devices and catheters, and it allows for the production of several-meter-long samples. Furthermore, the silicone can be coextruded around a central fiber, easing the assembly process. However, several limitations remain, such as the impossibility of extruding silicones with low shore hardness and with millimetric or submillimetric dimensions. In this work, a 5 m long sample has been coextruded industrially^1^, around a central thread (Figure 3A), using a shore hardness 40A silicone (Table 1). The cross-section is shown in Figure 3B. It can be observed that the cross-section is not perfectly circular and that the wall thickness is critically reduced between the central channel and two pressurized channels. No adhesion between the central thread and the silicone was observed. Small segments of 50 mm in length have been cut and reinforced using the same protocol as presented in Section 3.1. They are characterized in Section 4 along with the molded prototypes. To demonstrate the usability of the actuator, a 90 cm long steerable catheter is described in Section 5 and is made of a softer molded active segment assembled with a long extruded main body.

## 4 Characterization

To understand the capabilities of the designed pneumatic actuator, a complete experimental characterization is performed. The mechanical capabilities (curvature and blocking force) are measured, and the stiffness influence is further studied. Furthermore, the working space, multi-channel actuation, and dynamic capabilities are also investigated.

### 4.1 Mechanical Characterization

Euler–Bernoulli beam theory can provide a first interpretation of the working principle of the actuator (Xavier et al., 2021; Gorissen et al., 2017; Decroly et al., 2020). It can be considered that the pressure *p* generates a moment *M* ∼ *p*, constant along the length *L* of the actuator, which causes the bending of the actuator with a constant curvature *k* and an angle *α*: $\frac{\alpha }{L_{0}}=k=\frac{1}{R_{c}}=\frac{M}{EI_{z}}$ . Contrarily to the angle *α*, the radius of curvature *R*_*c*_ = *l*/*α* and the curvature *k* = 1/*R*_*c*_ are independent of the length *L*. The bending is inversely proportional to the bending stiffness of the actuator, which depends on the material (*E*) and geometrical properties (*I*_*z*_) of the actuator. Euler–Bernoulli beam theory relies on several unverified assumptions. We consider 1) small deformations, 2) that the cross-sections remain to the neutral fiber and undistorted, and 3) that the material is linear elastic. Here, strains up to 25% are estimated during actuation, deformation of the cross-section is observed when pressurizing the actuator due to the inflation of the circumferential channels, and the silicone is known to be hyperelastic (Marechal et al., 2020). Despite these limitations, this model allows for a deeper understanding and can be used to generalize the results and compare the developed actuator to that in the literature.

**FIGURE 4:**
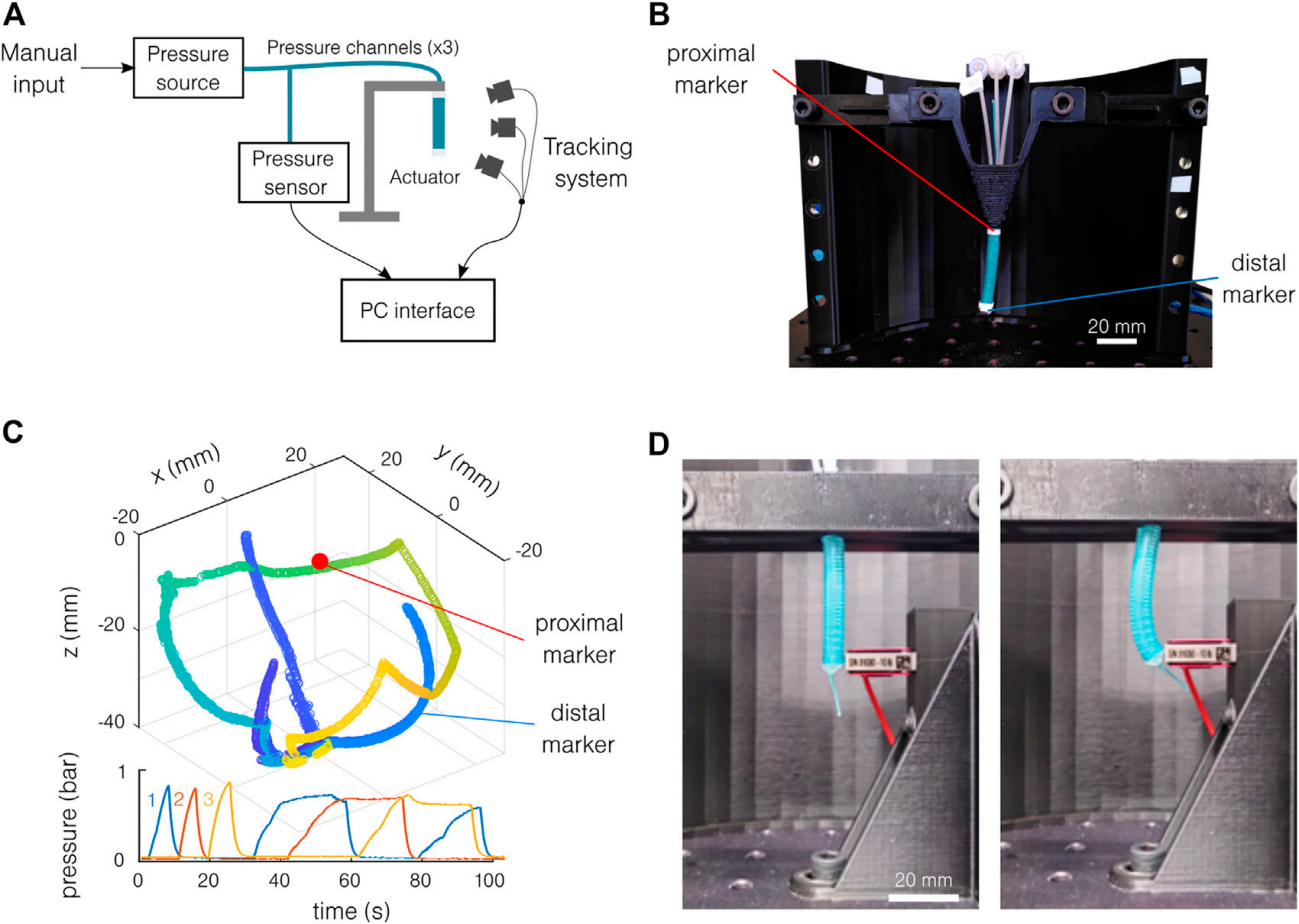
Experimental setup. **(A)** Schematics of the motion tracking setup. **(B)** The actuator is placed vertically, and the position of the two markers is tracked. **(C)** Typical motion tracking results with the corresponding pressure pattern. The color in the spatial representation indicates the time. The three colors on the pressure graph indicate the different channels. **(D)** Setup for blocking force measurement, at rest (left), and actuated (right).

The bending characterization test bench is composed of a custom pressure controller and the OptiTrack Motive infrared capture system^2^. The pressure controller is inspired by the Soft Robotics Toolkit^3^ and documented under a Creative Commons Attribution-NonCommercial 4.0 International License^4^. The actuator is placed vertically on a support (Figure 4A). Two infrared reflective markers are placed on the sample (Figure 4B): a semi-sphere *end marker* on its extremity and a cylindrical *fixed marker* as closest as possible to the fixation. Three additional markers are placed on the support for redundancy. The same pressure pattern is repeated for each test and shown in Figure 4C with the tracking results. The three channels are first pressurized individually, and then each combination of channels is tested, making the actuator do a full rotation around the vertical axis. Two samples of each material are used, tested five to six times, with a length varying between 20 and 30 mm. The movement of the sample compared to the workspace of the tracking system is relatively small, and the measurement precision is limited by the markers. The markers tracking can be lost, and a jump in the end marker position can sometimes be observed. This limits the precision of the setup to the order of magnitude of the radius of the actuator in the worst case (3 mm). The curvature is given by the curvature radius of a circle tangent to the *z*-axis and containing both the end marker and the fixation position. These three conditions lead to the circle center coordinates and the targeted radius.

The force measurement setup is similar to the bending characterization setup. A precision force sensor (Futek LSB205^5^) is used instead of the motion tracking system. The sample is placed vertically, fixed at its proximal end, and tangent to the force sensor at its distal end (Figure 4D). Two samples are tested for each material. The pressure is increased and decreased four times in a row. The tests are repeated for each channel and with two free lengths: 18.8 and 33.8 mm. If the actuator is not perfectly placed in the bending direction, the blocking force will be underestimated. Note that the error on the bending direction is estimated to be 15°, giving an error of 3.5% of the projected force (cos(15°) = 0.965). Furthermore, the exact free length of the actuator is only estimated. The exact fixation of the actuator is estimated at the bottom of the support. The point of action of the force is estimated at the middle of the force sensor height.

**FIGURE 5:**
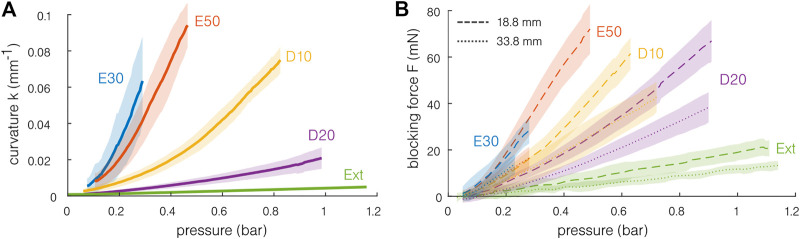
Quasi-static mechanical capabilities of the actuator for different materials. The shaded region indicates the standard deviation. **(A)** The softest actuators achieve a curvature of above 0.07 mm^−1^ for pressure inferior to 1 bar. A curvature of 0.1 mm^−1^ corresponds to a radius of curvature of 10 mm and to a bending angle of approximately 180° for an actuator of 30 mm. **(B)** The blocking force is measured for two different free lengths (18.8 and 38.8 mm). The maximal blocking forces remain inferior to 100 mN. The stiffer the material, the lesser the force exerted for a given pressure.

The measured curvature is presented as a function of the pressure for single-channel actuation in Figure 5A. As expected, the softer materials achieve a stronger bending at a given pressure. For all materials except the Dragon Skin 20 and the extruded silicone, curvatures higher than 0.05 mm^−1^, corresponding to radii of curvature smaller than 20 mm, can be achieved repeatedly and without bursting, at pressures below 1 bar. Note that, for the same materials, it was also possible to reach a radius of curvature close to or lower than 10 mm before reaching the samples’ pressure limits. This corresponds to a bending angle of approximately 180° for an actuator of 30 mm. The actuation of the Dragon Skin 20 and the extruded silicone was limited by the pressure source. The samples seem to be able to support higher pressures. Note that small hysteresis can be observed for the softer materials, having larger curvatures for a given pressure when decreasing it. Similar behavior is obtained when measuring the blocking force of the actuator. No strong difference is observed between the samples for a given material, demonstrating good test repeatability. Consequently, the fabrication imprecision and differences seem to be negligible considering the bending capabilities of the samples.

The mean blocking force results are presented in Figure 5B. Forces of 20–80 mN can be obtained. Larger blocking forces are reached with the shortest sample. This is coherent with a simple Euler–Bernoulli model of a beam subjected to a moment *M*. The moment and the blocking force *F* are linked by the relation *M* = *FL*_0_, with constant *M* for a given pressure. At a given pressure, the blocking force is higher for the softest material. This can be understood from the fact that the stiffer material required more energy to deform, inducing a smaller force transmission with the pressure.

**FIGURE 6:**
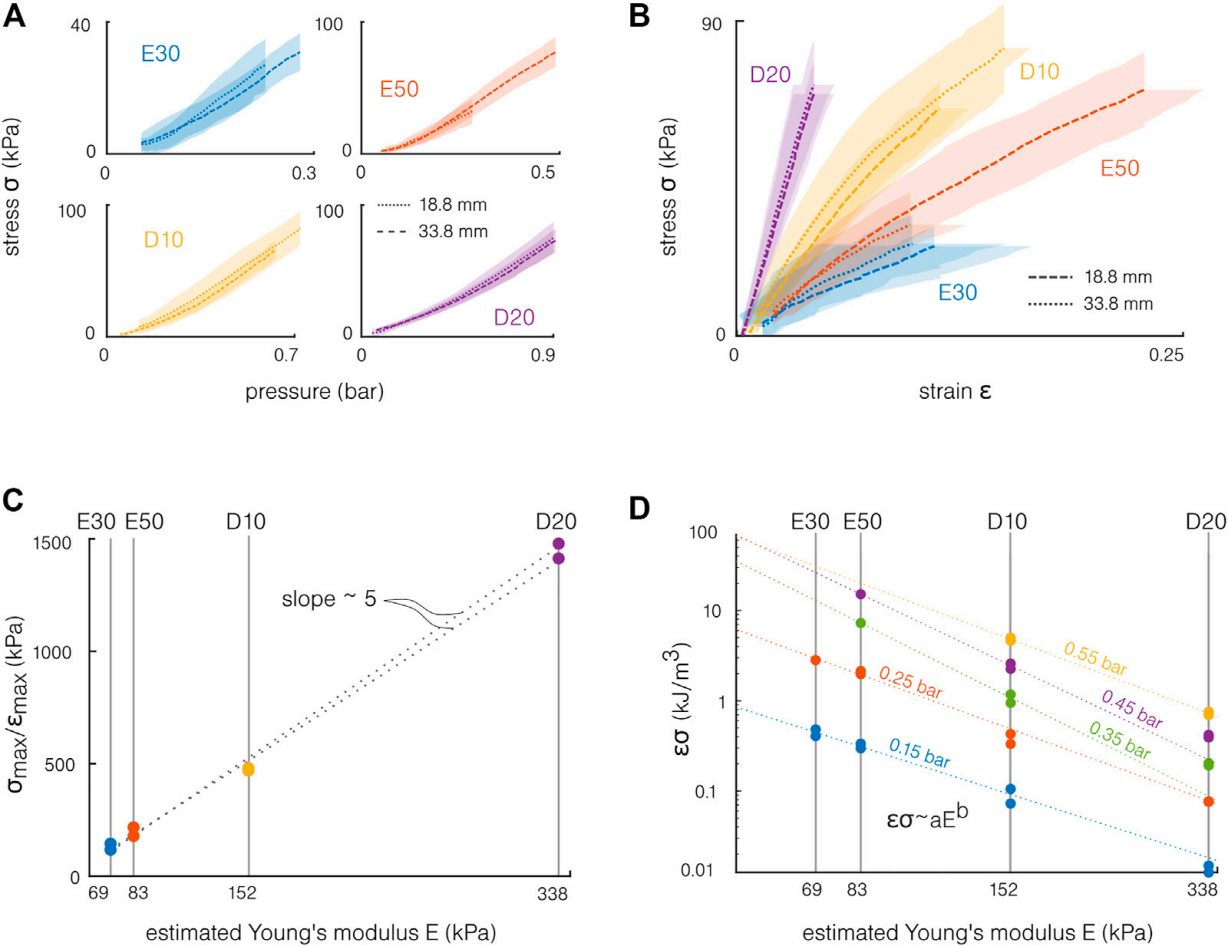
Influence of the material for the molded actuators. **(A)** The actuation stresses computed from the blocking force superpose for the two tested lengths. **(B)** The actuation stress and strain are synchronized using the pressure. A stiffer material increases the slope of this curve, “stiffening” the actuation. **(C)** The actuation stiffness *σ*_*max*_/*ϵ*_*max*_ is proportional to the material stiffness. **(D)** An estimated energy density *σϵ* is presented in a log–log diagram for different pressures. This product increases with the pressure and decreases with the material stiffness. The experimental values can be fitted by the equation *σϵ* = *aE* ^*b*^, with *a* and *b* fitting parameters.

The results can be further analyzed to generalize the actuators’ characterization, to compare it to that in the literature, and to study further the material influence. This analysis is based on several assumptions and, in particular, on the assumption of a constant curvature, which is a strong assumption, although common (Xavier et al., 2021). The extruded material is not included in this study, since no estimated Young’s modulus is provided. Under the Euler–Bernoulli beam theory, the actuation strain can be obtained from the most elongated fiber of the actuator. Note that as developed before, the hypothesis underlying this model is not verified, except for very low pressures. The distance between the neutral fiber and the most elongated fiber *d* corresponds here to the actuator radius, i.e., 3 mm: $\epsilon \equiv \frac{L-L_{0}}{L_{0}}=\frac{\alpha d}{L_{0}}=kd$ . The actuation strain is then considered directly proportional to the curvature *k*. Similarly to the strain, actuation stress can be obtained from the blocking force. The actuator can here be considered a beam exerting a force *F* perpendicular to its extremity. This force is generated by the constant moment *M* due to the pressure. Similarly to the strain, the actuation stress can be obtained in the most constrained fiber of the actuator: $\sigma \equiv \frac{Md}{I_{z}}=\frac{FL_{0}d}{I_{z}}$ . This presents the advantage to normalize the results—theoretically being independent of the actuator length. The mean stress as a function of the pressure for different materials is presented in Figure 6A. The stress curves superpose for all materials, meaning that the hypothesis of a constant moment *M* is well verified.

The bending and force capabilities can be synthesized by synchronizing the stress and strain on the pressure. The actuation stress–strain curves for the different materials are presented in Figure 6B. The strain is computed as *ϵ* = *kd*. Following this simple model, the maximal strain remains below 25%, which is relatively small compared to the maximal elongation of the materials. At these “low strains,” the datasheet with estimated Young’s modulus values provides a sufficient description of the material behavior. Note that higher strain appears locally, and the previous work highlighted the need of considering the material’s hyperelasticity when modeling soft silicone actuators (Marechal et al., 2020). Similarly, the maximal experimental stress remains one order of magnitude lower compared to the silicones’ tensile strengths (Table 1). This difference can be explained first by the stress concentrations and the simple model limitations, but also by the experimental setup. Even if the risk of burst was non-negligible for the Ecoflex samples, the maximal stress was also limited by the pressure source’s maximal pressure and by the risk of slipping off the force sensor for the sample. Note that this representation combines two different situations: the actuation strain is measured in free bending (no force) and the actuation stress from the blocking force (no displacement). However, this allows providing a first understanding of the material stiffness influence. The slopes correspond to the ratio between the actuation strain and the actuation stress, which corresponds to an “actuation stiffness”: *E*_*act*_ = *σ*/*ϵ* (Huber et al., 1997). The slope increases with the material hardness, meaning that, at a given pressure required to achieve a given curvature, the blocking force will be higher for stiffer materials. The first approach linear elastic model indicates that the actuation stress should be equal to the material Young’s modulus: $E_{act}=\frac{\sigma }{\epsilon }=\frac{Mr}{I_{z}}/\frac{Mr}{EI_{z}}=E$ . However, the actuation stiffness is around five times higher than the estimated Young’s modulus. This is confirmed in Figure 6C, which presents the actuation stiffness as a function of the estimated Young’s modulus for the maximal experimental pressure of each material.

Finally, a maximal energy density can also be estimated from the actuation stress *σ* and strain *ϵ*: $\sigma \epsilon =\frac{Mr}{I_{z}}\frac{Mr}{EI_{z}}={\left(\frac{Mr}{I_{z}}\right)}^{2}/E^{\left(-1\right)}$ (Huber et al., 1997). Note that this energy density is overestimated. The energy density should be computed as the same product, but considering the developed stress at the given strain. This energy density is represented as a function of the estimated Young’s modulus in Figure 6D, for different pressures. By analogy with the model, the experimental results can be fitted by the dashed lines *σϵ* ∼ *aE* ^*b*^, with *a* and *b* fitting parameters depending on the pressure, and *b* < 0. As described previously, the results do not follow exactly the model but give a deeper understanding of the material influence. The fact that the energy density decreases with the material stiffness can be interpreted as follows: the energy conversion (from pressure to motion or force) is more efficient for the softest materials. It can also be observed that the energy density increases as expected with the pressure.

Toward the design of the actuators, a general rule is that the softest material will allow the best energy conversion, i.e., the highest conversion of the pressure into stress and strain. However, this should not be the only criterion for choosing a material: stiffer materials can be used with higher pressures, allowing them to reach equivalent bending capabilities. Furthermore, the actuation stiffness increases strongly with the material stiffness. The stiffer the material, the higher the developed force at a given curvature.

### 4.2 Working Space

Following the assumption of constant curvature *k* along the length of the actuator *L*, the end marker moves on a surface of revolution supported by a parametric curve of equation:

$$\left\{\begin{array}{c} r=\frac{1-\cos\left(kL\right)}{k},\;\\ z=\frac{-\sin\left(kL\right)}{k}.\; \end{array}\right.$$

**FIGURE 7:**
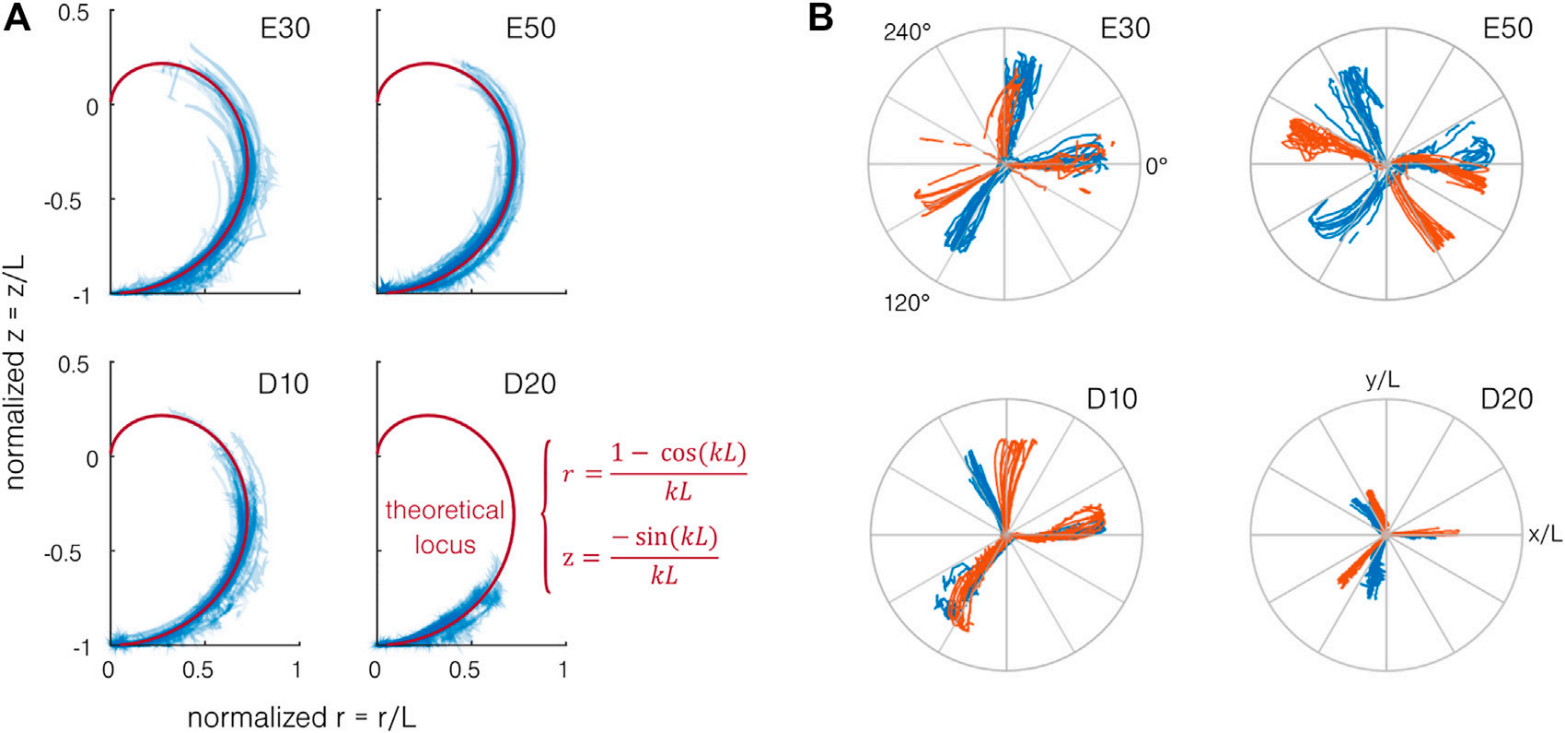
Working space of the actuator. **(A)** When projected on a vertical plane *Orz*, the end position of the end marker of the actuator (in blue) follows the theoretical locus (in red) for each material. **(B)** When projected on the *Oxy* plane and normalized by the actuator length, the position of the end marker shows a large difference from the expected 120° separation of the bending directions for each channel. Two actuators per material are represented, each color indicating one actuator. Normalized top view of the end marker position for each sample. The two colors indicate the two samples.

The normalized theoretical locus and experimental data point projected on a single vertical plane are presented in Figure 7A. Theoretically, the end marker should follow the locus while staying in the same *Orz* plane when increasing the pressure in a single channel. By actuating multiple channels simultaneously, it is possible to revolve around the vertical axis *z*. As observed in Figure 7A, the end marker follows the theoretical locus relatively well, validating the constant curvature hypothesis. However, differences up to 10% can be observed between the experimental and theoretical loci, mainly for the multi-channel actuation. Those differences could be explained by the setup limitations (error on the marker position and the estimated fixation location and actuator axis). The normalized position in the horizontal plane *Oxy* of the end marker during the first part of the test is presented in Figure 7B. Contrarily to the bending capabilities, we can observe a strong difference between the samples. Given the actuator design, the bending traces should be separated by 120° for each channel, which is not the case for several samples. Furthermore, a small *hook* can be observed at high pressure for several samples. This behavior can be explained by fabrication limitations. If the 120° angle between the channels is not perfectly respected, 1) the bending direction is modified and 2) the wall thickness becomes uneven, leading to 2.a) uncontrolled anisotropy and 2.b) risks of leakage. Despite those imperfections, as stated before, the same curvature can be achieved for the different samples, and multi-channel actuation allows to reach the same working space.

### 4.3 Multi-Channel Actuation

**FIGURE 8:**
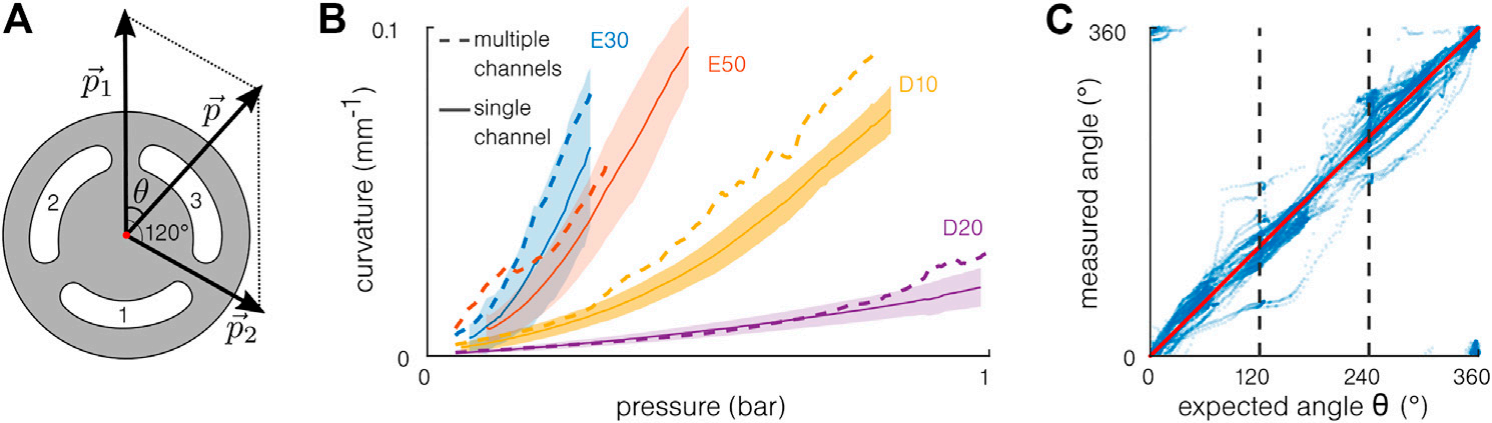
Multi-channel actuation. **(A)** Model for multi-channel actuation: the pressures ${\vec{p}}_{1}$ and ${\vec{p}}_{2}$ result in a pressure $\vec{p}$ having an angle *θ*. **(B)** Experimental results of curvature in multi-channel actuation using the computed combined pressure and compared to those of the single-channel actuation. The model underestimates the pressure required to reach a given angle in multi-channel actuation. **(C)** The bending direction follows the model and the expected angle *θ* in multi-channel actuation, despite some large variations.

The multi-channel actuation results are obtained from the second part of the test when two channels are actuated simultaneously. Figure 8A illustrates a simple model developed to estimate the combined driving pressure. The channels are considered separated from 120°, and the pressure ${\vec{p}}_{i}$ in the channel *i* induces bending in the direction opposed to the channel position. Summing those vectors allows to compute a *combined* driving pressure $\vec{p}$ . The curvature achieved with multi-channel actuation is compared to that with single-channel actuation in Figure 8B as a function of the pressure. Interestingly, the curvature obtained with multi-channel actuation and corrected with the pressure combination model is higher than the results obtained with single-channel actuation. The fabrication limitations should not be the cause of this difference here because they do not influence strongly the bending capabilities. Furthermore, averaging the samples would have compensated for the differences. Consequently, the difference is probably explained by the large deformation of the cross-section. Indeed, to reach a given combined pressure in multi-channel actuation, the sum of the pressure is still way higher than that in single-channel actuation, leading to larger internal deformations of the cross-section, and the model is too limited to capture. The actual direction of bending *θ* is represented as a function of the direction predicted by the model in Figure 8C. The reference angle (*θ* = 0) is defined as the bending direction of the first channel. Only the data points with large bending are plotted. The bending direction results seem to validate the model: despite some deviations, the experimental results follow the model. The deviations are observed mainly when one channel dominates the bending (*θ* close to 0°, 120°, or 240°), and the samples presenting the most deviation are the same as in Figure 7B. The differences could then be explained by fabrication imprecision.

### 4.4 Dynamic Capabilities

**FIGURE 9:**
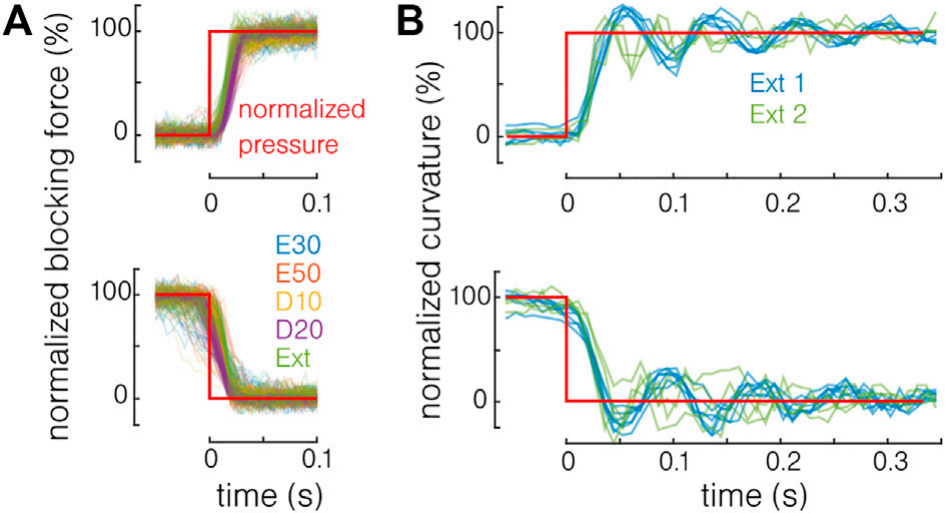
Response time of the actuator. **(A)** In blocking force, no difference is observed between the different materials. **(B)** In bending, the response time remains in the same order of magnitude.

The dynamic capabilities of the actuator are also investigated. The same setup presented in Section 4 is used. Constant pressure is delivered by the pressure source, and a three-way valve that is opened and closed manually is added just before the actuator. The characterization is performed in the blocking force (using the Futek LSB205 force sensor with an acquisition rate of 300 samples per second and with a free length of 18.8 mm) and free bending, using a camera (120 frames per second) and using image processing. The results are shown in Figure 9. The blocking force (Figure 9A) and curvature (Figure 9B) are normalized by the maximal value of each test to compare the different materials and actuation pressures. Two actuators for each material have been tested in force and two extruded actuators in bending. No strong difference is observed between the different materials in force. The bending response is only tested with extruded actuators. Even if in the same order of magnitude, the response in force is quicker (<0.05 s) than in bending (<0.3 s) because the actuator deformation is limited. It is also expected that the absolute curvature value has an influence on the response time in bending. The results are similar to previously reported pneumatic actuators’ response time (Gorissen et al., 2018) and are not limited toward the development of endoscopic tools, the surgical motion frequency being generally comprised below 2 Hz (Hu et al., 2006). However, toward the development of integrated systems, the pressure supply and pressure leads will be predominant in determining the response time of the system (Joshi and Paik, 2020).

## 5 Use-Case and Demonstration

**FIGURE 10:**
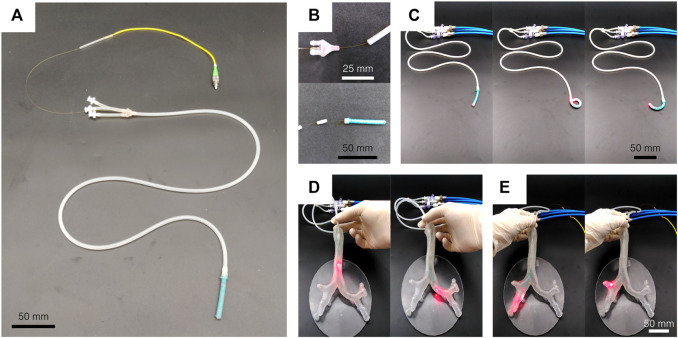
Steerable catheter demonstrator. **(A)** A soft active tip is assembled on an 85 cm long extruded segment, and an optical fiber is placed in the working channel. **(B)** 3D printed connectors are used to assemble the segments. **(C)** The actuator is still able to bend in every direction and to reach high curvature. **(D)** The steerable catheter can be inserted in a bronchial tree phantom and allows to navigate by bending the tip in the desired direction. **(E)** The tertiary bronchi of the right upper lobe can easily be illuminated by the light transmitted by the optical fiber.

While showing promising results, the developed actuator remains far from any clinical application. In this section, a 90 cm long steerable catheter using the developed actuator is presented (Figure 10A). This demonstrator is made by the assembly of an 85 cm long extruded catheter without reinforcement and a 50 mm long actuator made of Dragon Skin 10. Small rigid connectors are 3D printed to connect the pressure leads of the different segments in Figure 10B. Silicone glue is used for sealing. For the demonstration purpose, a 0.45 mm diameter multi-mode optical fiber is inserted in the working channel of the steerable catheter to replace the thread and act as a neutral fiber. Toward endoscopic applications, a camera or another device could be placed at the tip of the catheter, and its electrical leads or optical fibers are inserted similarly in the working channel. As shown in Figure 10C, when pressurized, only the softer Dragon Skin 10 active tip bends and still achieves high curvature. The higher stiffness of the extruded body prevents it from bending. See the Supplementary Video for a complete demonstration. The same pressure source as in Section 4 is used. A bronchial tree phantom (Cheng et al., 2016) has been 3D printed to demonstrate the potential of the solution for navigation. In Figure 10D, the demonstrator is inserted in the left main bronchus, by pressurizing the opposite channel at the bifurcation of the trachea. Figure 10E shows that the tertiary bronchi of the right upper lobe can easily be illuminated by the light transmitted by the optical fiber.

## 6 Discussion

The main results of this work are compared with those in the literature in Table 2. This non-exhaustive comparison presents recent pneumatic actuators intended for minimally invasive surgery. Although it uses saline as the working fluid, the microcatheter developed by Gopesh et al. (2021) is included in the comparison as a reference catheter that has reached the stage of *in vivo* study in porcine. The presented design is similar to other pneumatic actuators in terms of diameter, length, number of channels, and mechanical capabilities. However, our design presents the advantage of combining three pressurized channels and a working channel, with a diameter as small as 6 mm.

**TABLE 2:** Comparison of pneumatic actuators for MIS and endoscopy.

	External diameter (mm)	Actuated length (mm)	Actuation time (ms)	Maximal pressure (bar)	Minimal radius of curvature (mm)	Maximal blocking force (mN)	Main material stiffness (MPa)	Number of channels	Fabrication process	Working channel	MIS device demonstrated
Suzumori et al. (1997)	16	48		4	30.5*		1.2*	3	Extrusion	No	No
Ikuta et al. (2012)	3	25	50–200*	5	8.5*	50		1	Molding	Yes	Two-module assembly demonstrated for blood vessel navigation in Ikuta et al. (2003)
Sun et al. (2016)	6	50–80		1	5		0.07*	3	Molding—reinforcement	Yes	No
Gorissen et al. (2018)	1	10	11	2.8	3.5*		1*	1	Molding	No	3 cm long device*, with demonstration on ophthalmological phantom
Abidi et al. (2018)	14.5	50		1.5	16.7*		0.08*	3 × 2	Molding—reinforcement—module assembly	Yes	10 cm long device with two actuated modules, with demonstration of laparoscopic total mesorectal excision on a cadaver
Garbin et al. (2019)	13.5	6			5.7*		7.1	3	Commercial module assembly	Yes	120 cm long device, with demonstration of endoscopic stomach visualization on pigs and a cadaver
Zhang et al. (2019)	15	65		0.13	23.2*	450	0.08*	3	Molding—reinforcement	Yes	No (assembly of three modules shown)
Zhang et al. (2019)	3.5						0.08*	3	Molding	Yes	No
Manfredi et al. (2019)	18	20			8.8*		0.07*	3	Molding—reinforcement—module assembly	Yes	6 cm long device with one actuated module and two balloons, with demonstration of colonoscopic navigation on phantom
Decroly et al. (2020)	5	16–48		0.35	8.5	42.9	0.07*	1	Molding—reinforcement	Yes	No
Chauhan et al. (2021)	8.5	30–40		0.45	13.1*		0.15*	3	Molding	Yes	No
Gopesh et al. (2021)**	0.9	15	<1	3.5	2.5		<0.151	4	Molding/assembly	Yes	160 cm long endovascular neurosurgical catheter tested in an *ex vivo* silicone model and *in vivo* porcine
This work	6	20–50	50–300	0.3–1.2	10–50	20–80	0.07–0.6*	3	Overmolding/extrusion—reinforcement	Yes	120 cm long device, with illustration on a lung phantom

* indicates estimated values.

**Gopesh et al. (2021) use saline as the working fluid but include the microcatheter in the comparison as a reference catheter that has reached the stage of *in vivo* study in porcine.

Furthermore, the innovative fabrication processes described in this work allowed to easily implement this design with different silicones and efficiently add a reinforcement layer using silicone-soaked thread. The extrusion process reported in this work is very promising, both for developing reinforced segments and to act as the main body of steerable catheters. However, the geometry reported in this work pushed the limits of the technology in terms of miniaturization and silicone softness.

The contribution of this work also lies in the complete characterization performed. Large bending can be obtained (curvature up to 0.1 mm^−1^ for Ecoflex 50 actuators, corresponding to a bending angle of approximately 180° for a length of 30 mm). The exerted force remains below 100 mN, and with no rigid parts in the design, it limits the risks of damage on surrounding tissues when used in endoscopic configurations. Furthermore, the response time of the actuator has been shown not limited for endoscopic applications and independent of material stiffness. Finally, despite fabrication errors influencing strongly the bending direction in single-channel actuation, the complete working space can still be achieved by multi-channel actuation. The simple multi-channel actuation model presented in this work showed limitations to capture the complete behavior of the actuator because of the large deformations of the cross-section and its sensibility to the fabrication error.

The influence of stiffness has also been thoroughly investigated based on the experimental results, and using a simple Euler–Bernoulli model, relying however on the strong assumption of constant curvature. It showed that the value of stress at 100% elongation can provide a good first approximation to describe the material influence on the bending and blocking force capabilities of the studied design. Toward the design of the actuators, a general rule is that the softest martial will allow the best energy conversion, i.e., the highest conversion of the pressure into stress and strain. Furthermore, the stiffer the material, the stiffer the developed force at a given curvature.

Finally, we demonstrate that the actuator can successfully be integrated into a complete steerable catheter design, able to carry optical fibers and to navigate in a bronchial tree phantom. This is an important first step toward future clinical applications of pneumatically actuated steerable catheters.

## 7 Conclusion

In conclusion, this work contributes to the development of a toolbox of soft robotic solutions for MIS and endoscopic applications, by 1) validating and characterizing a promising design, 2) describing versatile and scalable fabrication methods, 3) allowing for a better understanding of the influence of material stiffness on the actuator mechanical capabilities, and 4) demonstrating the usability of the solution in a potential use-case.

However, toward clinical application, it will be necessary to start from the specific requirements of a given application to use this toolbox wisely, by adapting and completing the steerable catheter presented here. In particular, concerning the material stiffness, the softness can be beneficial to ensure the patient safety and achieve large deformations. However, this can be a limitation for endoscopic procedures, to 1) exert forces, 2) push the tool into remote locations, and 3) carry a stiffer tool than an optical fiber. Integration of stiffness variation solutions will likely be required to enhance the catheter capabilities and reach clinical applications (Blanc et al., 2017; Gifari et al., 2019). Furthermore, other solutions for the integration of the actuated segment in the overall steerable catheter could be investigated, to discard the need for rigid connectors and additional assembly steps. For example, varying the reinforcement pattern and the fiber angle on a fully extruded catheter could be promising, to limit locally the bending (Connolly et al., 2017). Finally, developing a finite element model could allow for a finer prediction of the performance and optimization of the design for specific applications (Xavier et al., 2021).

## Data Availability

The raw data supporting the conclusions of this article will be made available by the authors, without undue reservation.

## Footnotes

1 Primasil Silicones Ltd.

2 2021 NaturalPoint, Inc., DBA OptiTrack, Beaverton, OR, United States

3
https://softroboticstoolkit.com/book/control-board

4
http://fab.academany.org/2019/labs/ulb/students/gilles-decroly/projects/pressure-control.html

5 FUTEK Advanced Sensor Technology, Inc., Irvine, CA, United States

6 Ecoflex™ Series, Smooth-On, Inc., Dragon Skin™ Series, Smooth-On, Inc., PR410/40, Primasil Silicones Ltd.
